# Phototherapy improves cognitive function in dementia: A systematic review and meta‐analysis

**DOI:** 10.1002/brb3.2952

**Published:** 2023-04-05

**Authors:** Xinlian Lu, Chengyu Liu, Feng Shao

**Affiliations:** ^1^ School of Psychological and Cognitive Sciences Peking University Beijing P. R. China; ^2^ Department of General Surgery, Beijing Hospital, National Center of Gerontology Institute of Geriatric Medicine, Chinese Academy of Medical Sciences Beijing P. R. China

**Keywords:** behavioral and psychological symptoms of dementia, cognitive function, dementia, phototherapy, sleep

## Abstract

This study aimed to investigate the effectiveness of phototherapy intervention on cognitive function in older adult patients with dementia. PubMed, Ovid MEDLINE, Web of Science, EMBASE, Cochrane Central Registry of Controlled Trials, PsycINFO, and Clinical Trials were searched from their inception to August 10, 2022, for randomized controlled trials involving patients with dementia who received phototherapy interventions. We used the weighted mean difference (MD) or standard weighted mean difference to generate the pooled estimates. The primary outcome was cognitive function as measured by the Mini‐Mental State Examination (MMSE) score. The secondary outcomes were the behavioral and psychological symptoms of dementia (BPSDs) and sleep. This systematic review and meta‐analysis was registered in PROSPERO (registration number: CRD42022343788). We included 12 randomized controlled trials comprising 766 patients with dementia (426 patients in the intervention group and 340 in the control group). Phototherapy interventions significantly improved MMSE scores (*n* = 3, MD 2.68, 95% confidence interval [CI]: 1.38–3.98, *I* ^2^ = 0%). There were no significant differences in the Cornell Scale for Depression in Dementia score, Cohen‐Mansfield Agitation Inventory score (MD: −3.12, 95% CI: −8.05, 1.82, *I* ^2^ = 0%), Neuropsychiatric Inventory score, sleep efficiency, total sleep time, and Sleep Disorders Inventory score between the groups. Our systematic review and meta‐analysis showed that phototherapy significantly improved cognitive function in patients with dementia.

## Graphical abstract

This systematic review and meta‐analysis showed that phototherapy did not improve the behavioral and psychological symptoms of dementia significantly but improved cognitive function in older patients with dementia.

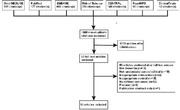

## 1 INTRODUCTION

Dementia is characterized by progressive and severe cognitive impairment, motor deficits, and behavioral problems (Aarsland, 2020). As a major health problem in the elderly (Zucchella et al., 2018), dementia can lead to individual dependence, disability, and even death (Malik et al., 2022). It not only affects the quality of life of individuals, but also imposes enormous social and economic burdens on families, health systems, and society (Aranda et al., 2021). With the aging population worldwide, dementia has become an important public health problem and there is a desperate need for effective and low‐cost treatment (Cibeira et al., 2020; Prince et al., 2015).

As drug treatment for dementia has limitations such as medical contraindications, limited efficacy, and adverse effects (Azhar et al., 2022; Wong, 2016), non‐pharmacological therapy has been increasingly regarded as a critical part of comprehensive dementia care (Li et al., 2021; Sink et al., 2005). Phototherapy, which utilizes full‐spectrum bright light usually above 600 lux (Onega et al., 2016; Zou et al., 2022) or wavelength‐specific lights, such as blue‐enriched (Cremascoli et al., 2021) or blue‐green (Nowak, 2008) lights, is a promising non‐pharmacological therapy that has the advantages of non‐invasiveness, inexpensive, and high safety (Forbes et al., 2014; C.‐R. Liu et al., 2021; Scales et al., 2018).

While most phototherapy‐related studies have focused on sleep, with high heterogeneity among the interventions and studied populations, reported results remain inconsistent (Forbes et al., 2014). The effect of phototherapy on cognitive function, the decline of which is the clinical hallmark of dementia (Kales et al., 2014), and behavioral and psychological symptoms of dementia (BPSDs), which significantly impact patients’ quality of life and distresses caregivers (Bessey & Walaszek, 2019), still need to be clarified. Therefore, we present this systematic review and meta‐analysis to examine the effects of phototherapy on cognitive function, BPSDs, and sleep in older adult patients with dementia.

## 2 MATERIALS AND METHODS

This systematic review followed the Preferred Reporting Items for Systematic Reviews and Meta‐Analysis (PRISMA) statement guidelines (The PRISMA checklist is shown in Table S1). The protocol for this review was registered with PROSPERO (CRD42022343788).

### 2.1 Eligibility criteria

Randomized controlled trials (RCTs) that investigated phototherapy interventions in elderly patients with dementia were eligible for this review. The primary outcome of concern was cognitive function, which was assessed via the Mini‐Mental State Examination (MMSE) (Folstein et al., 1975). The MMSE is the most commonly used assessment tool to measure global cognitive function (Bos et al., 2015). It is a 30‐point questionnaire that assesses simple tasks in many areas, including memory, working memory, orientation, language, and visuospatial abilities, with lower MMSE scores indicating more severe cognitive impairments.

The secondary outcomes were as follows: BPSDs (such as agitation, aberrant motor behavior, anxiety, irritability, depression, and apathy) and sleep disturbances. The BPSDs were mainly measured using the Cornell Scale for Depression in Dementia (CSDD) (Alexopoulos et al., 1988), Cohen‐Mansfield Agitation Inventory (CMAI) (Cohen‐Mansfield & Billig, 1986), and Neuropsychiatric Inventory (NPI) (Cummings, 1997). Measurements of sleep disturbances included total sleep time (TST), sleep efficiency (SE), and sleep disorder inventory (SDI) (Tractenberg et al., 2003).

The CSDD consists of 19 items that evaluate the frequency of symptoms related to depression within the preceding week. Symptoms are grouped into five main domains, including “Mood‐related signs,” “Behavioral disturbance,” “Cyclic functions,” “Physical signs,” and “Ideational disturbance.” A high score indicates a depressive state. The CMAI is a 29‐item scale that evaluates a series of abnormal behaviors with particular emphasis on agitation. A higher score represents more severe behavior. The NPI is a reliable and valid assessment of the behavioral and psychological symptoms of patients with dementia. Twelve neuropsychiatric symptoms are evaluated: delusions, hallucinations, depression, anxiety, disinhibition, agitation/aggression, euphoria/exaltation, apathy/indifference, irritability/lability, motor disorder, appetite disorders, and sleep. Each symptom is scored based on its severity and frequency. A higher score indicates more severe symptoms.

TST refers to the total amount of sleep time, and SE refers to the percentage of time spent asleep in the rest interval. These two objective sleep parameters are usually detected using actigraphy and are commonly used to measure sleep‐wake rhythms (Cremascoli et al., 2021). The SDI was completed by nursing staff to assess seven symptoms related to sleep disturbances and scored in terms of severity, frequency, and caregiver distress.

The inclusion criteria were as follows: (1) elderly adults with dementia, including Alzheimer's disease (AD), vascular dementia (VD), dementia with Lewy bodies (DLB), Parkinson's disease with dementia (PDD), mixed dementia (MD), or dementia due to other causes; (2) phototherapy interventions; (3) one of the above‐defined outcomes; (4) controls that received no intervention but only routine care or health education; and (4) RCT study design. Given that the phototherapy intervention studies in patients with dementia were all implemented in elderly adults, we did not set the age limit at the time of the search.

The exclusion criteria were as follows: (1) population that included mild cognitive impairment (MCI), (2) control group that received a phototherapy intervention, (3) inaccessible full‐text article, and (4) secondary analysis.

### 2.2 Search strategy

We used the keyword searching with the terms “dementia,” “phototherapy,” and “cognition” in PubMed, Ovid MEDLINE, Web of Science, EMBASE, Cochrane Central Registry of Controlled Trials (CENTRAL), PsycINFO, and Clinical Trials, searching from inception to August 10, 2022. The full search strategy for PubMed is shown in Table S2. When available, subject headings (such as Medical Subject Headings) and filters (such as RCT) were combined to search the databases.

### 2.3 Study selection

Two reviewers (Xinlian Lu and Chengyu Liu) independently assessed the records for eligibility and resolved any disagreements through discussion. RCTs that compared the effects of phototherapy on cognitive function in patients with dementia were included. The patients were diagnosed with dementia, including AD, VD, DLB, PDD, MD, or dementia due to other causes, according to any accepted criteria, such as those of the Diagnostic and Statistical Manual of Mental Disorders (DSM‐IV, DSM‐V). The outcome of cognitive function must have been subjectively and objectively measured, and the intervention duration was included.

### 2.4 Study quality

Two reviewers (Xinlian Lu and Chengyu Liu) independently assessed the study quality by evaluating the bias risk using the Cochrane Collaboration's tool. Bias risk was assessed using the following criteria: (1) double blinding of participants and personnel, (2) generation of random sequence, (3) allocation of concealment, (4) blinding of outcome assessment, (5) selective reporting, (6) incomplete outcome data, and (7) other biases.

### 2.5 Data extraction

Two reviewers (Xinlian Lu and Chengyu Liu) independently extracted all data. The collected data were as follows: (1) country, age, and sex; (2) countries where RCTs were implemented and a cognitive function assessment tool was used; (3) phototherapy intervention data (including frequency, type, intensity, and time) and intervention duration; and (4) values of outcome measures, such as cognitive function, sleep quality, emotion, and quality of life. We extracted the data using Excel and resolved disagreements through discussion.

### 2.6 Data synthesis and analysis

One reviewer (Xinlian Lu) initially completed the data synthesis process using Review Manager (RevMan) Version 5.4.1 (the Cochrane Collaboration), and another reviewer (Chengyu Liu) checked the data profile later. This meta‐analysis focused only on continuous variables; therefore, we used the weighted mean difference (MD) or standard weighted mean difference (SMD) to generate pooled estimates. If *I* ^2^ ≥ 50%, a random‐effects model was used, and if *I* ^2^ < 50%, a fixed‐effects model was chosen. The *I* ^2^ statistic was used to present statistical heterogeneity; we also performed a post hoc sensitivity analysis and exploration of publication bias.

## 3 RESULTS

### 3.1 Search results

**FIGURE 1:**
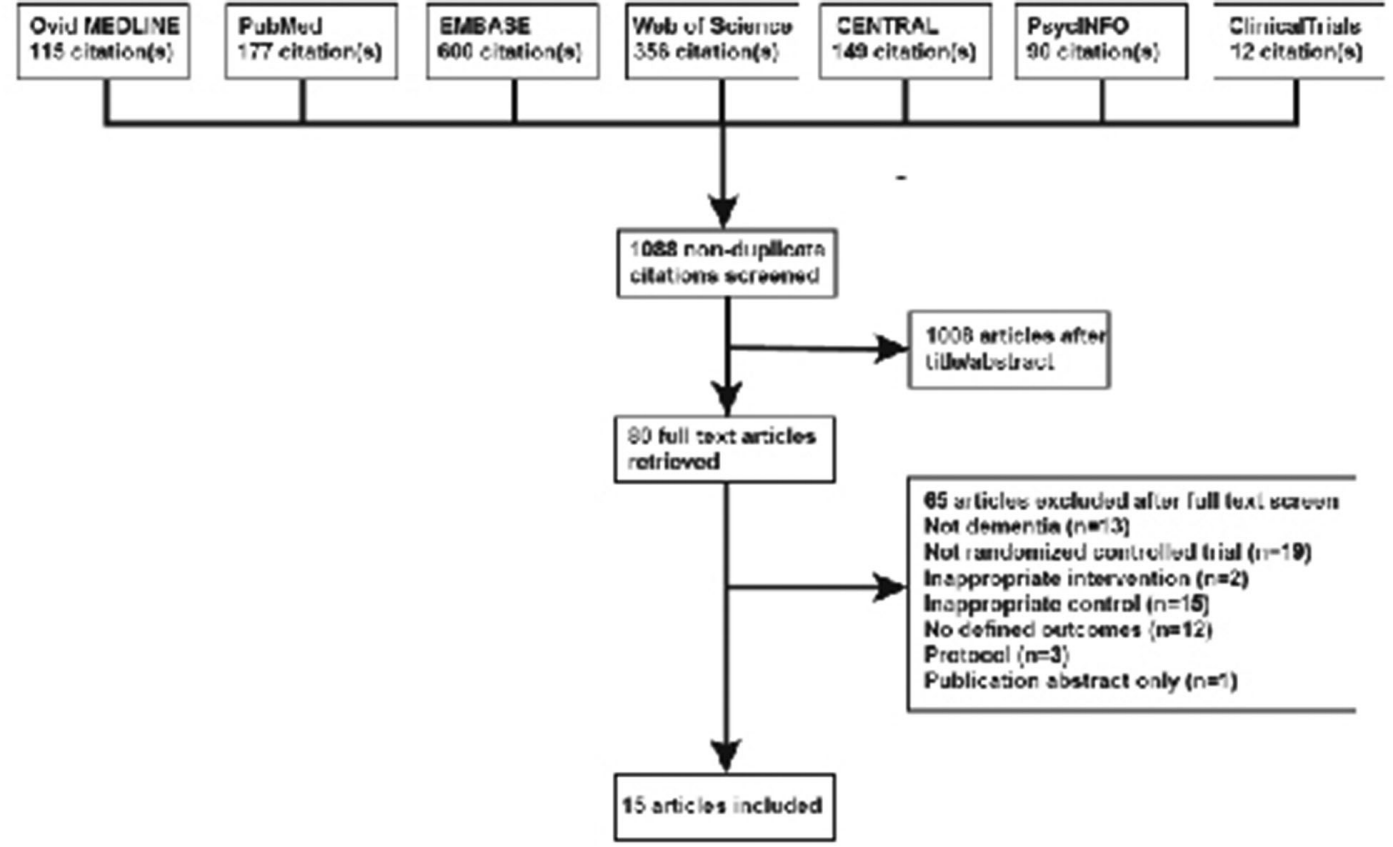
Preferred Reporting of Systematic Reviews and Meta‐Analyses flow diagram.

We screened 1088 citations according to our search strategy (Figure 1). Sixty‐five studies were excluded from the full‐text evaluation of 80 articles because the patient population (*n* = 13), intervention (*n* = 2), study type (*n* = 19), comparison group (*n* = 15), study design (*n* = 3), publication abstract only (*n* = 1), or outcomes (*n* = 12) did not meet the inclusion criteria. Ultimately, 15 articles were included. It should be noted that Kolberg et al. (2021) and Hjetland et al. (2021) reported on the same trial, and Dowling et al. (2007), Dowling, Hubbard, et al. (2005), and Dowling, Mastick, et al. (2005) reported on the same trial; therefore, these 15 articles only described 12 studies.

### 3.2 Baseline characteristics

We included 766 patients with dementia (intervention group, *n* = 426; control group, *n* = 340) (Table 1). Among all participants, the proportion of women was higher. At least nine of the 12 studies included over 50% women, as one study (Graf et al., 2001) did not mention the sex ratio, two studies (Cremascoli et al., 2021; Nizamutdinov et al., 2021) included more men, and one study (Nowak, 2008) included only female patients. The mean age of these patients ranged from 73.73 to 85.9 years old. Four studies (Cremascoli et al., 2021; Figueir et al., 2019; Kolberg et al., 2021; Nowak, 2008) used the DSM‐IV or DSM‐V to diagnose dementia, one study (Zou et al., 2022) used the International Classification of Diseases (ICD‐11) diagnostic standards, one study (Dowling et al., 2007) used the National Institute of Neurological and Communication Disorders and Stroke/Alzheimer's Disease and Related Disorders Association(NINCDS‐ADRA) criteria, four studies (Fontana Gasio et al., 2003; Graf et al., 2001; McCurry et al., 2011; Nizamutdinov et al., 2021; Onega et al., 2016) confirmed dementia using doctors’ diagnoses or medical records, and two studies (Burns et al., 2009) used other tools to diagnose dementia. Among these patients with dementia, five studies (Cremascoli et al., 2021; Dowling et al., 2007; McCurry et al., 2011; Nowak, 2008; Zou et al., 2022) only included patients with Alzheimer's disease, and the remaining seven studies included several types of dementia, such as AD, VD, DLB, PDD, and MD.

**TABLE 1:** Characteristics of included studies of phototherapy on older patients with dementia

		Groups patients number			Age, year, M ± SD			Female, *n* (percentage)					Phototherapy interventions							
Study	Country	IG	CG	Total	IG	CG	Total	IG	CG	Total	Dementia assessment method	Type of dementia	Frequency	Intensity	Type	Time	Duration	Co‐interventions	Control	Different visits
Zou et al. (2022)	China	34	27	61	75.94 ± 9.47	73.04 ± 9.34	/	18 (52.9%)	15 (55.6%)	33 (54.1%)	ICD‐11	AD	Once a day	14000 lux	Bright light	30 min, from 9 a.m. to 9:30a.m.	4 Weeks	N/A	50 lux dim light	2 Week, 4 week
Nizamutdinov et al. (2021)	USA	40	20	60	72.4 ± 8.2	77.8 ± 5.2	74.2 ± 7.7	41%	47%	/	Doctors’ diagnoses or medical records	Dementia	Twice a day	Wavelength of 1060–1080 nm and 15,000 mW	Helmet devices	6 min	8 Weeks	N/A	The same hamlet without light	8 Week
Kolberg et al. (2021) Hjetland et al. (2021)	Norway	33	36	69	84.3 ± 6.2	82.8 ± 7.9	83.5 ± 7.1	25 (75.8%)	22 (61.1%)	47 (68.1%)	DSM‐V	AD, some unknown or other dementia type	Everyday	400‐1000 lux light sequence on daytime, 100 lux on nighttime	Bright light (LED light panels)	24 h	24 Weeks	N/A	150–300 lux standard indoor light	8 Week, 16 week, 24 week
Cremascoli et al. (2021)	Italy	8	5	13	72.23 ± 2.44	75.73 ± 3.81	73.37 ± 2.40	2 (25%)	2 (40%)	4 (30.1%)	DSM‐IV	AD	Once a day	10,000 lux	Blue‐enriched light (Luminette)	20 min	4 Weeks	N/A	50 lux dim light	4 Week
Figueiro et al. (2019)	USA	25	21	46	/	/	85.3 ± 7.7	/	/	30 (65.2%)	DSM‐IV	ADRD	Everyday	550–700 lux	Bright light (light box, light panels)	24 h (tailored lighting)	14 Weeks	N/A	110–200 lux dim light	5 Week, 14 week
Onega et al. (2016)	USA	30	30	60	/	/	82.6 ± 9.6	/	/	43 (61.7%)	Doctors’ diagnoses or medical records	Dementia	Twice a day, five day per week	10000 lux	Bright light (Light box)	30 min	8 Weeks	N/A	250 lux dim light	8 Week
McCurry et al. (2011)	USA	34	33	67	80.6 ± 7.3	81.2 ± 8.0	/	19(56%)	17(51%)	36(53.7%)	Doctors’ diagnoses or medical records	AD	Everyday	2500 lux	Bright light (light box)	60 min	8 Weeks	N/A	Daily routine	8 Week, 24 week
		33	33	66	80.0 ± 8.2	81.2 ± 8.0	/	20(61%)	17(51%)	37(56.1%)								NITE‐AD	Daily routine	
Burns et al. (2009)	England	22	26	48	84.5 ± 7.97	81.2 ± 8.0	/	16(73%)	16(62%)	/	A diagnosis of dementia (WHO, 1993)	AD,VD, DLB, MD	Everyday	10000 lux	Bright light	2h (10 a.m.–12 p.m.)	2 Weeks	N/A	100 lux dim light	4 Week, 8 week
Nowak (2008)	USA	10	10	20	/	/	85.9 ± 6.24	10 (100%)	10 (100%)	20 (100%)	DSM‐IV	AD	Everyday	12000 lux	Blue‐green light	30 min (between 6 a.m. and 7 a.m.)	2 Weeks		5 lux dim red light	2 Week, post 15/29/43 day
Dowling et al. (2007) Dowling, Hubbard, et al. (2005) Dowling, Mastick, et al. (2005)	USA	29	17	46	/	/	84 ± 10	/	/	57(81.4%)	NINCDS‐ADRDA	AD	Five times per week	Above 2500 lux	Bright light (natural light, light box)	1 h (9:30a.m.–10:30a.m.)	10 Weeks	N/A	150–200 lux	10 Week
		24	17	41	/	/		/	/							1 h (3:30 p.m.–4:30 p.m.)		N/A	150–200 lux	10 Week
Fontana et al. (2003)	Switzerland	9	4	13	86.8 ± 4.1	83 ± 4.5	85 ± 5	9 (100%)	3 (75%)	12 (92.3%)	Doctors’ diagnoses or medical records	AD, VD, DLB, PDD	Everyday	0.001 lux‐200 lux in the dawn, 200 lux‐0.001 lux in the dusk	Bright white light	1 h (15 min in the dawn,45 min in the dusk)	3 Weeks		5 lux dim red light	3 Week
																				6 Week
Graf et al. (2001)	Austria	13	10	23	78.8 ± 8.4	85.8 ± 4.5	81.8 ± 7.6	/	/	/	Doctors’ diagnoses or medical records	AD, VD	Once a day	3000 lux	Bright light (light box)	2 h (5 p.m.–7 p.m.)	10 Days		100 lux dim light	10 Day

Abbreviations: AD, Alzheimer's disease; ADRD, Alzheimer disease and related dementias; CG, control group; IG, intervention group; DSM‐IV/V, the Diagnostic and Statistical Manual of Mental Disorders; MD, mixed dementia; ICD‐11, the International classification of diseases; NINCDS‐ADRDA, The National Institute of Neurological and Communicative Disorders—Alzheimer's Disease and Related Disorders Association; NITE‐AD, Nighttime Insomnia Treatment and Education in Alzheimer's Disease; NITE‐AD, DLB, dementia with Lewy bodies; PDD, Parkinson's disease with dementia; RCT, randomized controlled trial; VD, vascular dementia.

### 3.3 Intervention characteristics

Studies that compared phototherapy interventions with control groups were included. Most (seven of 12) studies (Burns et al., 2009; Dowling et al., 2007; Figueir, 2019; Graf et al., 2001; Kolberg et al., 2021; McCurry et al., 2011; Onega et al., 2016) employed routine daily light in the control group, other studies (Cremascoli et al., 2021; Fontana Gasio et al., 2003; Nizamutdinov et al., 2021; Nowak, 2008; Zou et al., 2022) utilized dim light (≤50 lux) or devices without light.

Phototherapy interventions of all forms, frequencies, and durations were included in this review. In most (eight of 12) studies (Burns et al., 2009; Dowling et al., 2007; Figueir, 2019; Fontana Gasio et al., 2003; Graf et al., 2001; McCurry et al., 2011; Onega et al., 2016; Zou et al., 2022), phototherapy intervention was implemented using bright light, while two (Kolberg et al., 2021; Nizamutdinov et al., 2021) studies used LED light, and the remaining two (Cremascoli et al., 2021) used blue or blue‐green light. Phototherapy duration generally ranged from 6 to 120 min. Half (six of 12) of the studies implemented phototherapy interventions at specific times of day, three (Burns et al., 2009; Nowak, 2008; Zou et al., 2022) in the morning, one (Graf et al., 2001) in the afternoon, one in (Fontana Gasio et al., 2003) the dawn‐dusk period, and one (Dowling et al., 2007) consisting of two intervention groups in the morning and afternoon. Besides, four studies (Cremascoli et al., 2021; McCurry et al., 2011; Nizamutdinov et al., 2021; Onega et al., 2016) did not report a specific intervention time, and two (Figueir, 2019; Kolberg et al., 2021) utilized a 24‐h lighting sequence. The frequency ranged from twice a day to five times per week, while most (nine of 12) studies used phototherapy once a day.

### 3.4 Adverse events

Among 12 RCTs including 426 patients received phototherapy intervention, four patients were reported to have mild adverse events (Burns et al., 2009; Cremascoli et al., 2021; Graf et al., 2001; Nowak, 2008). Two patients developed symptoms of ocular mild irritation (Cremascoli et al., 2021; Graf et al., 2001). One patient had slight redness on the forehead, which was relieved about half an hour, and completely recovered the next day (Nowak, 2008). In the Burns et al. (2009) trial, one patient dropped out after 3 days of intervention because the treatment reminded her of torture memories during World War II. Kolberg et al. (2021), Nizamutdinov et al. (2021), and Zou et al. (2022) clearly reported that no participants experienced adverse events after the phototherapy. The remaining seven trials did not mention adverse events associated with phototherapy.

### 3.5 Risk of bias

Of the 12 RCTs, three studies showed a low risk of bias, five studies showed a high risk of bias in one to three domains, and bias results in the remaining studies were unclear due to the lack of details on selection bias (Figures S1 and S2).

### 3.6 Impact of interventions

#### 3.6.1 Cognitive function

**FIGURE 2:**
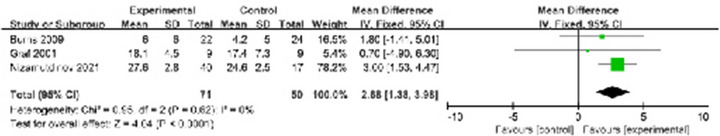
Mini‐Mental State Examination (MMSE) scores at 8‐week post‐intervention.

Significant positive intervention effects were observed for global cognitive function. A meta‐analysis of three RCTs (Burns et al., 2009; Graf et al., 2001; Nizamutdinov et al., 2021) involving 121 participants found that phototherapy had a positive effect on post‐intervention MMSE scores, which differed significantly between the experimental and control groups (MD: 2.68, 95% CI: 1.38–3.98, *I* ^2^ = 0%) (Figure 2).

3.6.2

No significant differences were found in the intervention effects on depression symptoms. Four RCTs (Burns et al., 2009; Figueir, 2019; Kolberg et al., 2021; Onega et al., 2016) reported CSDD scores. There were no significant differences between the scores between groups (MD: −0.70, 95% CI: −3.10 to 1.70; *I* ^2^ = 81%) (Figure S3).

**FIGURE 3:**
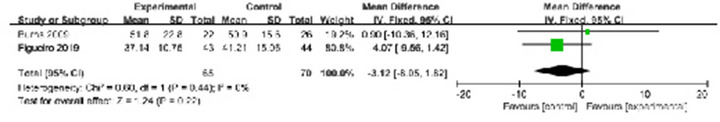
The Cohen‐Mansfield Agitation Inventory (CMAI) scores.

Two RCTs (Burns et al., 2009; Figueir, 2019) reported CMAI scores, and no significant difference between groups was observed (MD: −3.12, 95% CI: −8.05 to 1.82; *I* ^2^ = 0%) (Figure 3). Higher CMAI scores indicated more severe agitation behaviors, and the results suggested that patients with dementia showed a trend of decreasing CMAI scores after the phototherapy intervention.

**FIGURE 4:**
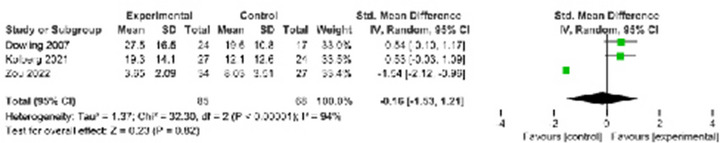
The Neuropsychiatric Inventory (NPI) scores.

The meta‐analysis of three trials (Dowling et al., 2007; Kolberg et al., 2021; Zou et al., 2022) comprising 153 participants found no significant differences in NPI scores between groups (SMD: −0.16, 95% CI: −1.53 to 1.21, *I* ^2^ = 94%) (Figure 4). As different versions of the NPI scale were used in these trials, we chose SMD rather than MD for the data analysis.

#### 3.6.3 Sleep

Three RCTs (Cremascoli et al., 2021; Kolberg et al., 2021; McCurry et al., 2011) reported TST, and no significant difference was observed between groups (MD: 6.42, 95% CI: −35.26 to 48.09, *I* ^2^ = 0%) (Figure S4).

**FIGURE 5:**
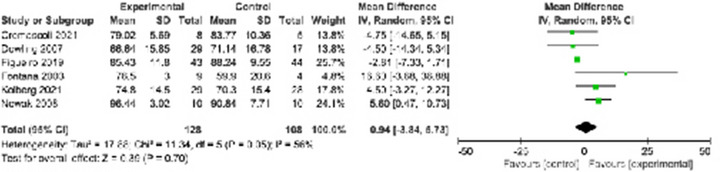
The total sleep time.

Six RCTs (Cremascoli et al., 2021; Dowling et al., 2007; Figueir, 2019; Fontana Gasio et al., 2003; Kolberg et al., 2021; Nowak, 2008) explored the effect of phototherapy on SE and found no significant difference in SE (MD: 0.94, 95% CI: −3.84 to 5.73, *I* ^2^ = 56%) (Figure 5).

SDI was evaluated in two RCTs (Kolberg et al., 2021; McCurry et al., 2011). The meta‐analysis concluded that phototherapy intervention had no significant effect on SDI (SMD: 0.15, 95% CI: −1.48 to 1.79; *I* ^2^ = 95%) (Figure S5).

## 4 DISCUSSION

Our meta‐analysis indicates that phototherapy improved cognitive function in patients with dementia but had no significant effect on BPSDs and sleep. This suggests that phototherapy may be one of the most promising non‐pharmacological interventions for improving core symptoms of dementia.

Several studies have supported the results of our meta‐analysis that phototherapy has a positive effect on cognitive function. A recent non‐randomized controlled pilot study (C.‐R. Liu et al., 2021) utilized 2500 lux bright light for 8 weeks in patients with mild‐to‐severe dementia and found significant improvement in the MMSE score from baseline to the 5th and 9th weeks compared with the control group. A classic long‐term RCT (Riemersma‐van der Lek et al., 2008) revealed that light reduced cognitive deficits in the elderly by 5%. It is important to note that this RCT was included in several meta‐analyses and systematic reviews which focused on phototherapy (Forbes et al., 2014, 2009; Mitolo et al., 2018; Tan et al., 2022), while not included in our review. For the purpose of observing the effects of phototherapy on patients with dementia, we only included trials of which all participants were diagnosed with dementia, while in this RCT, not all, only the majority (87%) of participants were. A previous meta‐analysis (Forbes et al., 2014) also showed a trend in improving cognitive function with phototherapy, but it was not statistically significant (MD: 1.24, 95% CI: −0.81 to 3.28; *I* ^2^ = 0%). This is quite different from our findings probably because we included a recent study (Nizamutdinov et al., 2021) and were more rigorous about the inclusion criteria for patients with dementia.

Studies have demonstrated that light may compensate for the reduction in the visual sensory input of patients with dementia and stimulate specific neurons in the suprachiasmatic nucleus of the hypothalamus to regulate circadian rhythms (Behrman et al., 2014; Hastings et al., 2018; LeGates et al., 2014; R.‐Y. Liu et al., 2000; Van Someren et al., 2002). As circadian rhythms are involved in optimal brain function (Walker & Stickgold, 2004), light supplementation may act on the synchronizing/phase‐shifting effects of circadian rhythms to improve cognitive function (An et al., 2020; Vandewalle et al., 2009).

A recent meta‐analysis showed the same results in sleep as our review, indicating no significant improvements in sleep efficiency (Tan et al., 2022). Previous meta‐analyses suggested that light had a negligible effect on CSDD scores, CMAI scores, NPI scores, and sleep efficiency. These results are consistent with our results and add to the reliability of our conclusions (Forbes et al., 2014, 2009).

As included RCTs reported only four patients experiencing mild adverse events, such as ocular or skin irritation, and turned to be transient, phototherapy seemed to be well tolerated and safe. We present suggestions for future research from the perspective of phototherapy devices. The light box is the most classic and commonly used device in phototherapy, and it provides full‐spectrum bright light usually over 2500 lux, with a duration of at least 30 min in the daytime, lasting 4–8 weeks (Fetveit et al., 2003; Graf et al., 2001; C.‐R. Liu et al., 2021; McCurry et al., 2011; Onega et al., 2016; Zou et al., 2022). It should be noted that the light box was placed 60 cm away from the patient at or above the patient's eye level. In addition, ceiling‐mounted light is a good choice for providing the designed whole‐day light in phototherapy. Compared with the light box, the installation cost of the ceiling‐mounted light is higher; however, the demand for staff is significantly lower and patients’ daily routines and treatment plans are minimally disrupted, which contribute to better compliance (Hjetland et al., 2021; Kolberg et al., 2021; van Hoof et al., 2012). In recent years, helmets and glasses have also been used as phototherapy devices, which usually employ light of a specific wavelength with a duration of approximately 15 min (Cremascoli et al., 2021; Nizamutdinov et al., 2021). Such portable devices allow for better control of light intensity and are ergonomic without interfering with patients’ normal activities.

In general, phototherapy appears to be a promising non‐pharmacological intervention without significant adverse effects. Therefore, further well‐designed studies are needed to explore the most effective clinical implementation conditions, including device type, duration, frequency, and time.

Our study had several strengths. This review focuses on the impact of phototherapy interventions on the core performance of cognitive function in patients with dementia. The review analyzed the effects of phototherapy intervention more comprehensively than previous meta‐analyses and adds to the evidence base in favor of the implementation of phototherapy interventions for patients with dementia.

This review had some limitations. First, most included studies did not report the value of the outcome change, we estimated the effect based on the post‐intervention outcome. Second, the number of RCTs that focused on the effects of phototherapy on patients with dementia was small, and several RCTs reported the following outcomes, such as interdaily stability and intradaily variability. These outcomes were not enough to conduct meta‐analysis. Third, the sample size was quite small, and some RCTs did not report disaggregated outcomes for patients with different types of dementia. Finally, due to the different types, frequencies, intensities, and durations of light interventions, the results included in this review were highly heterogeneous. Further studies are required to validate these methodologically heterogeneous results.

## 5 CONCLUSIONS

Our systematic review and meta‐analysis suggests that phototherapy is a promising intervention, as it can improve cognitive function in older patients with dementia.

## AUTHOR CONTRIBUTIONS

Xinlian Lu and Chengyu Liu performed data acquisition and screening. Xinlian Lu and Chengyu Liu participated in the data analysis. Xinlian Lu and Feng Shao were responsible for the conception and design of the study. Xinlian Lu, Chengyu Liu, and Feng Shao discussed the rationale and revised the manuscript accordingly. Feng Shao approved the final version of the manuscript for publication. All the authors discussed the results and made contributions to the manuscript.

## CONFLICT OF INTEREST STATEMENT

The authors declare no conflict of interest.

### PEER REVIEW

The peer review history for this article is available at https://publons.com/publon/10.1002/brb3.2952.

## Supporting information

Supporting Information

Supporting Information

Supporting Information

Supporting Information

Supporting Information

**Supplementary Table 1**. PubMed Search Strategy

Supporting Information

## Data Availability

All data are fully available without restriction.
