# Supplementary figures and images for: Phototherapy improves cognitive function in dementia: A systematic review and meta‐analysis
Source: Brain Behav. 2023 Apr 5;13(5):e2952. doi: 10.1002/brb3.2952 (PMC10176000; doi:10.1002/brb3.2952)

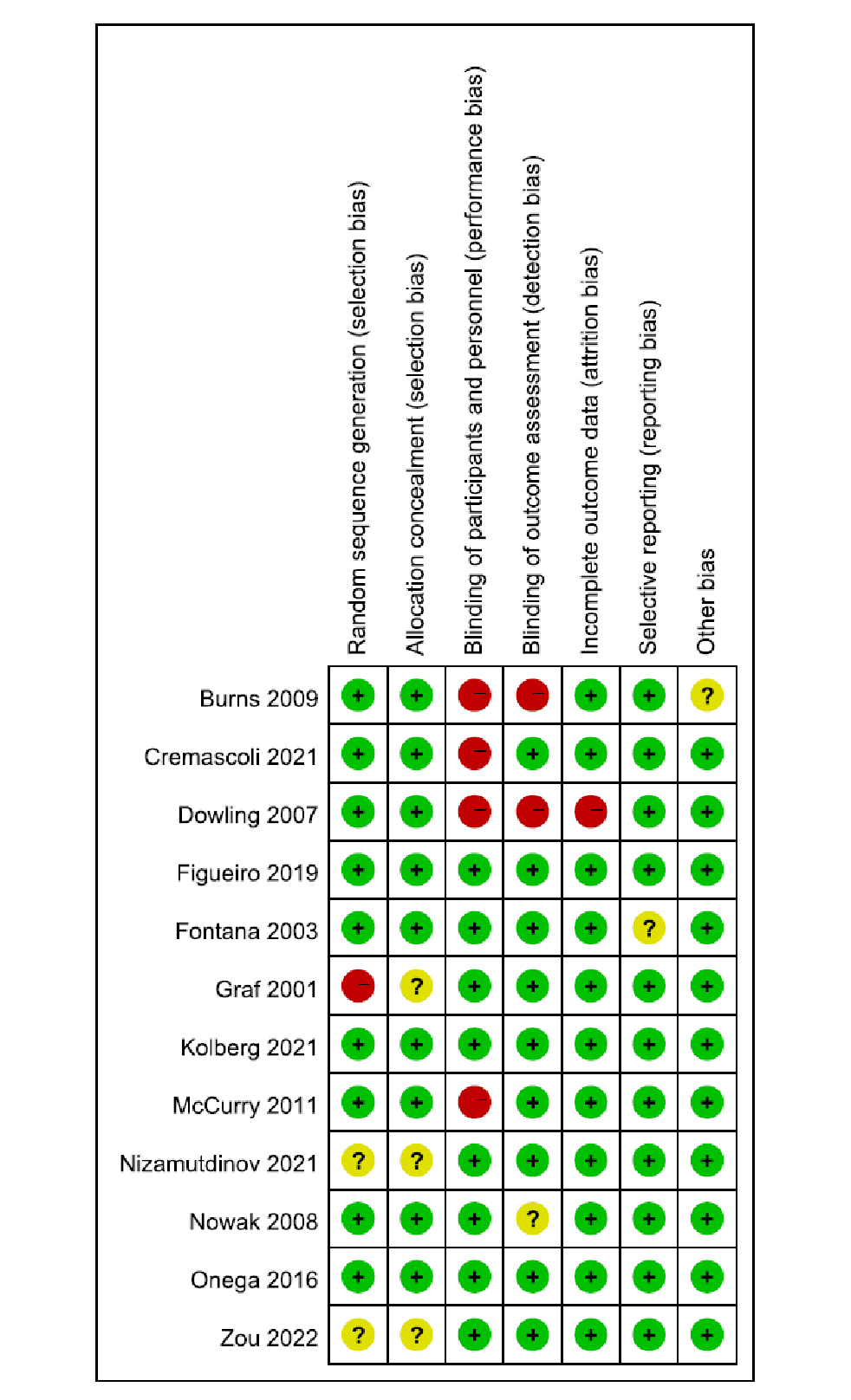

Supplement: Supplementary file 1 — Supporting Information [file BRB3-13-e2952-s003.jpg]

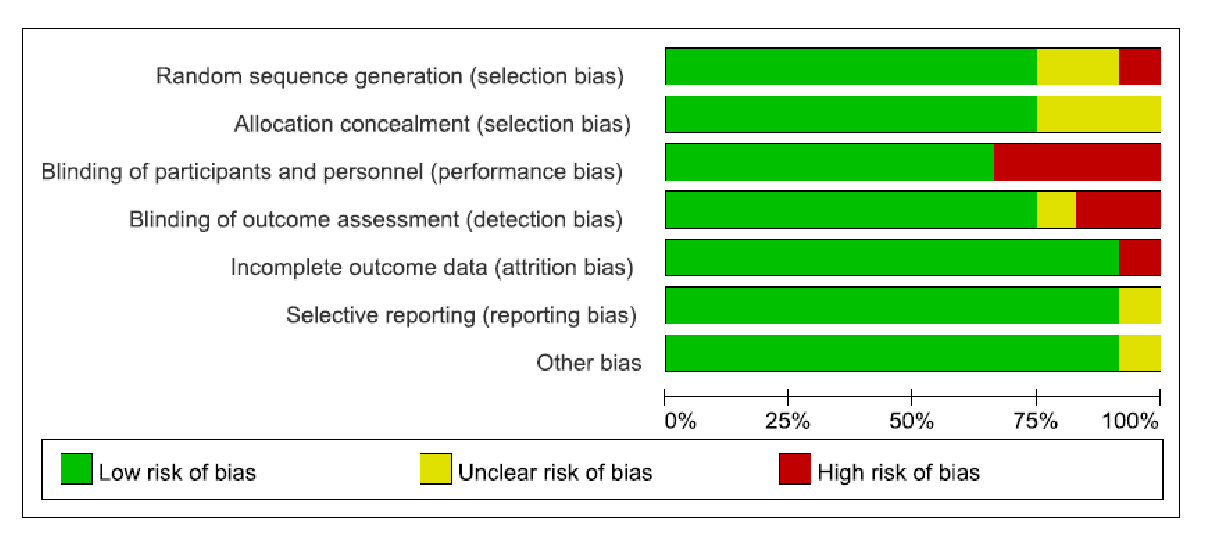

Supplement: Supplementary file 2 — Supporting Information [file BRB3-13-e2952-s001.jpg]

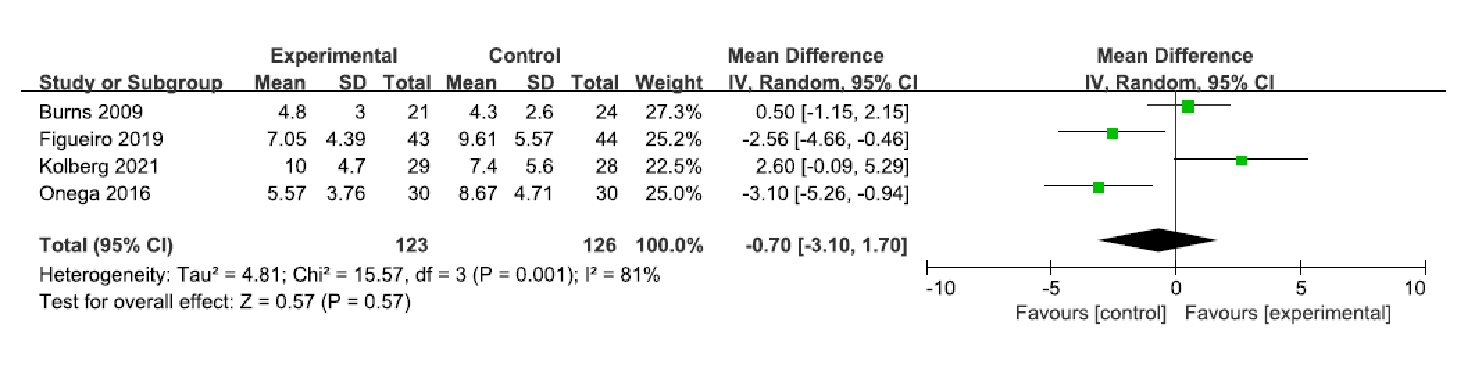

Supplement: Supplementary file 3 — Supporting Information [file BRB3-13-e2952-s002.jpg]

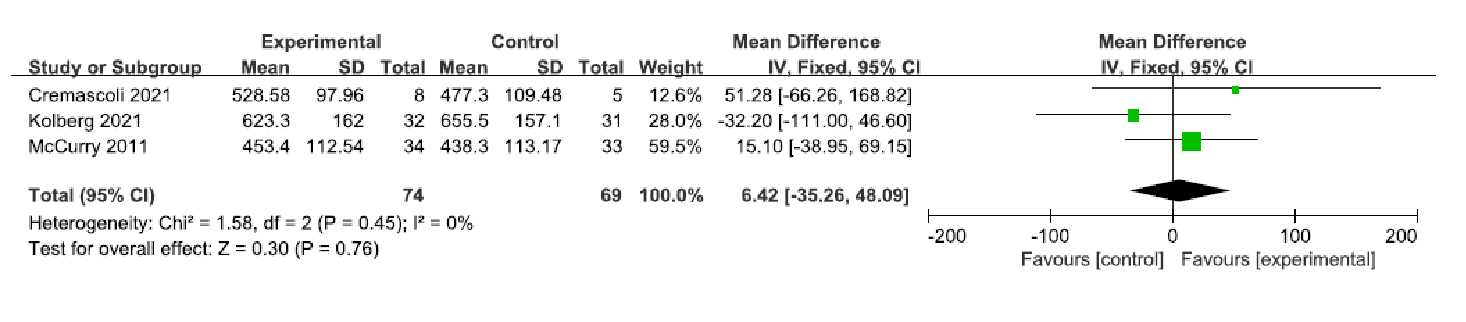

Supplement: Supplementary file 4 — Supporting Information [file BRB3-13-e2952-s005.jpg]

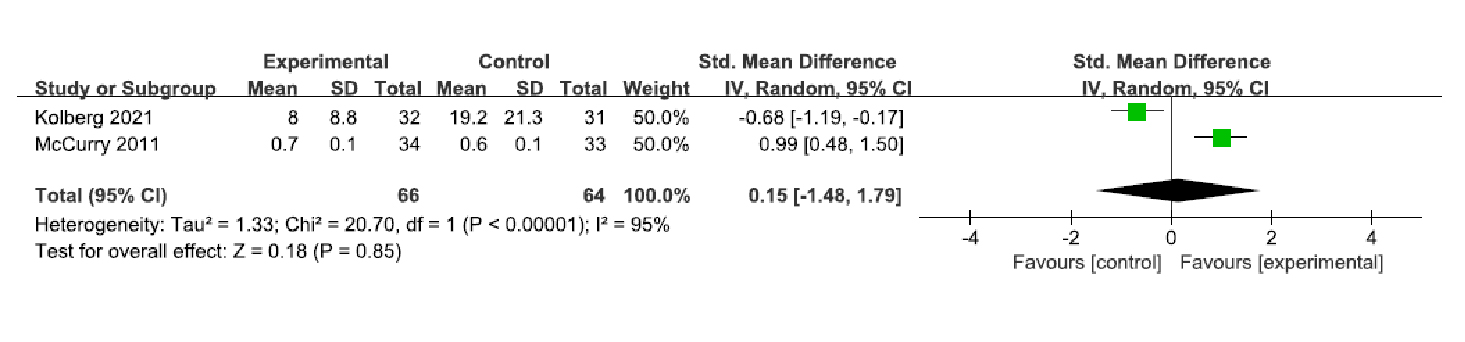

Supplement: Supplementary file 5 — Supporting Information [file BRB3-13-e2952-s006.jpg]
