# Supplementary material for: Phototherapy improves cognitive function in dementia: A systematic review and meta‐analysis
Source: Brain Behav. 2023 Apr 5;13(5):e2952. doi: 10.1002/brb3.2952 (PMC10176000; doi:10.1002/brb3.2952)
Supplement: Supplementary file 6 — Supplementary Table 1. PubMed Search Strategy [file BRB3-13-e2952-s007.docx]

**Supplementary Table 1.** PubMed Search Strategy

| **Database** | **Search Strategy** |
| --- | --- |
| Pubmed | 01: "Dementia"[Mesh] OR “Dementia, Vascular"[Mesh] OR "Alzheimer Disease"[Mesh] OR " Lewy Body Disease "[Mesh] OR “Parkinsonian Disorders” [Mesh] OR dementia with Lewy bodies[tw] OR Parkinsonian Dementia[tw]  02: "Aged"[Mesh] OR "Aged, 80 and over"[Mesh] OR "Frail Elderly"[Mesh] OR "Geriatrics"[Mesh] OR "Geriatric Psychiatry"[Mesh] OR "Geriatric Nursing"[Mesh] OR "Geriatric Dentistry"[Mesh] OR "Dental Care for Aged"[Mesh] OR "Health Services for the Aged"[Mesh]) OR (elder*[tw] OR eldest[tw] OR frail*[tw] OR geriatri*[tw] OR old age*[tw] OR oldest old*[tw] OR senior*[tw] OR senium[tw] OR very old*[tw] OR septuagenarian*[tw] OR octagenarian*[tw] OR octogenarian*[tw] OR nonagenarian*[tw] OR centarian*[tw] OR centenarian*[tw] OR supercentenarian*[tw] OR older people[tw] OR older subject*[tw] OR older patient*[tw] OR older age*[tw] OR older adult*[tw] OR older man[tw] OR older men[tw] OR older male[tw] OR older woman[tw] OR older women[tw] OR older female[tw] OR older population*[tw] OR older person*[tw]  03: "Dementia"[MeSH] OR Photo therapy[tw] OR light therapy[tw] OR light treatment[tw] OR bright light[tw] OR light box[tw] OR light visor[tw] OR heliotherapy[tw] OR dawn-dusk[tw] OR dawn dusk[tw] OR light*[tw]  04: "cognition"[Mesh] OR "memory"[MeSH] OR "sleep"[MeSH] OR "depression"[MeSH] OR "apathy"[MeSH] OR circadian rhythms[tw] OR mood[tw] OR attention[tw] OR agitation[tw] OR behavior[tw] OR neuropsychiatry[tw] OR language[tw] OR Visuospatial ability[tw] OR executive function[tw] OR personality[tw] OR sleep*[tw] OR depress*[tw] OR apath*[tw] OR agitat*[tw] OR behavior*[tw] OR behaviour*[tw] OR neuropsychiatr*[tw]  05: (clinical[tiab] AND trial[tiab]) OR "clinical trials as topic"[mesh] OR "clinical trial"[pt] OR random*[tiab] OR "random allocation"[mesh] OR "therapeutic use"[sh]  06: #1 AND #2 AND #3 AND #4 AND #5  Filter: Randomized Clinical Trial. |
